# Species Transferability of *Klebsiella pneumoniae* Carbapenemase-2 Isolated from a High-Risk Clone of *Escherichia coli* ST410

**DOI:** 10.4014/jmb.1912.12049

**Published:** 2020-05-21

**Authors:** Miyoung Lee, Tae-Jin Choi

**Affiliations:** 1 Department of Microbiology Pukyoung National University Busan 48513, Republic of Korea; 2 Department of Laboratory Medicine, BHS Hanseo Hospital Busan 48253, Republic of Korea

**Keywords:** *Escherichia coli* ST410, *Klebsiella pneumoniae* ST307, extraintestinal pathogenic *E. coli*, *blaKPC-2*, Tn4401a

## Abstract

Sequence type 410 (ST410) of *Escherichia coli* is an extraintestinal pathogen associated with multi drug resistance. In this study, we aimed to investigate the horizontal propagation pathway of a high-risk clone of *E. coli* ST410 that produces *Klebsiella pneumoniae* carbapenemase (KPC). blaKPC-encoding *E. coli* and *K. pneumoniae* isolates were evaluated, and complete sequencing and comparative analysis of blaKPC-encoding plasmids from *E. coli* and *K. pneumoniae*, antimicrobial susceptibility tests, polymerase chain reaction, multilocus sequence typing, and conjugal transfer of plasmids were performed. Whole-genome sequencing was performed for plasmids mediating KPC-2 production in *E. coli* and *K. pneumoniae* clinical isolates. Strains *E. coli* CPEc171209 and *K. pneumoniae* CPKp171210 were identified as ST410 and ST307, respectively. CPEc171209 harbored five plasmids belonging to serotype O8:H21, which is in the antimicrobial-resistant clade C4/H24. The CPKp171210 isolate harbored three plasmids. Both strains harbored various additional antimicrobial resistance genes. The IncX3 plasmid pECBHS_9_5 harbored *blaKPC-2* within a truncated Tn*4401a* transposon, which also contains *blaSHV-182* with duplicated conjugative elements. This plasmid displayed 100% identity with the IncX3 plasmid pKPBHS_10_3 from the *K. pneumoniae* CPKp171210 ST307 strain. The genes responsible for the conjugal transfer of the IncX3 plasmid included *tra/trb* clusters and *pil* genes coding the type IV pilus. ST410 can be transmitted between patients, posing an elevated risk in clinical settings. The emergence of a KPC-producing *E. coli* strain (ST410) is concerning because the *blaKPC-2*-bearing plasmids may carry treatment resistance across species barriers. Transgenic translocation occurs among carbapenem-resistant bacteria, which may spread rapidly via horizontal migration.

## Introduction

The gram-negative bacteria family *Enterobacteriaceae* includes pathogens that are responsible for a large number of infections and deaths worldwide each year. Furthermore, the continuously increase in the prevalence of antibiotic resistance in this bacteria family poses a serious problem [1]. Carbapenemases that endow bacteria with carbapenem resistance often jeopardize allopathic treatment for infectious diseases caused by common nosocomial pathogens. Currently, *Klebsiella pneumoniae* carbapenemases (KPCs), belonging to the Ambler class A carbapenemases, are the most clinically concerning enzymes because of their global presence and capability of using a broad spectrum of substrates, including most β-lactam drugs, except cephamycins [2, 3]. Molecular epidemiological studies on multilocus sequence typing (MLST) for KPC-producing *Escherichia coli* have revealed 131 more widespread strains than the ST410, ST69, ST93, ST167, ST354, and ST3948 strains based on their sequence type (ST) [4-7]. *E. coli* ST 410 has been reported worldwide as an extraintestinal pathogen and associated with resistance to fluoroquinolones, third-generation cephalosporins, and carbapenems. Recent studies on ten extended-spectrum β-lactamase (ESBL)-producing *E. coli* ST410 isolates from Germany indicated that this lineage includes a new successful clone with cross-sectorial transmission among wildlife, humans, pets, and animals used for commercial purposes through various environmental modes [8, 9]. The accumulation of multidrug resistance in *E. coli* ST410 over the past two decades, along with its potential for transmission between patients, indicates its high risk in clinical settings [10]. Previous studies on carbapenemase-producing *Enterobacteriaceae* (CPE) among inpatients demonstrated the clonal spreading of CPE between patients [11, 12]. The transposon Tn4401, which is based on highly mobile Tn3, facilitates the dissemination of *blaKPC* [13], potentially resulting in horizontal gene transfer to other bacterial species. As the mortality rate associated with invasive infections caused by CPE is high [14], the increasing incidence of carbapenem-resistant bacteria, which are resistant to nearly all antibiotics, is of great concern [15]. This study was performed to investigate the horizontal propagation pathway of the KPC-producing high-risk clone of *E. coli* ST410 in one patient. The mechanism and KPC delivery pathways based on the acquisition of multidrug resistance, including resistance to carbapenem, were investigated using whole-genome sequencing. This study elucidated the risk of this *E. coli* clone to spread genes involved in antibiotic resistance across species, providing clues that can be integrated in the management of high-risk clones in South Korea.

## Materials and Methods

### Patient description

A 79-year-old man with chronic kidney disease was admitted to a general hospital (Busan, Korea) on December 12, 2017, owing to kidney disease. Upon admission, a KPC-2 *E. coli* isolate (CPEc171209) was detected in rectal swabs, and on December 21, 2017, the 9th day of hospitalization, KPC-2 *K. pneumoniae* (CPKp171210) was isolated. This prospective study was conducted under the approval of the Institutional Review Board of the BHS Hanseo Hospital, Busan, Korea (approval number CTS-19-002).

### Bacterial Isolates and Antimicrobial Susceptibility Testing

We evaluated the clinical strains of *E. coli* (CPEc171209) and *K. pneumoniae* (CPKp171210) isolated from the rectal swabs harvested from the patient. Bacteria were identified through standard microbiological procedures and VITEK-2 (bioMérieux, Marcy-l’Etoile, France). The identification of all isolates was confirmed through 16S rDNA sequencing [16]. VITEK 2 AST N224 cards (bioMérieux) and disk diffusion tests on Mueller-Hinton agar (BD Biosciences, Franklin Lakes, NJ, USA) were used to determine antimicrobial susceptibilities in accordance with the Clinical and Laboratory Standards Institute (CLSI) guidelines [17]. The susceptibility test was conducted with ampicillin, aztreonam, cefotaxime, ceftazidime, cefoxitin, ciprofloxacin, amikacin, gentamicin, imipenem, meropenem, ertapenem, and colistin. The minimum inhibitory concentrations (MIC) for colistin were determined using the broth microdilution method with Mueller-Hinton broth (BD Biosciences) in accordance with the European Committee on Antimicrobial Susceptibility Testing guidelines [18]. The susceptibility results for tigecycline were confirmed through the E-test (bioMérieux).

For the modified carbapenem inactivation method (mCIM) test, organisms were incubated with a meropenem disk in tryptic soy broth (Bacto Laboratories, Mount Pritchard, Australia). For the ethylene diamine tetra acetic acid (EDTA)-mCIM (eCIM) test, EDTA was added to the broth to chelate metal ions required for metallo-β- lactamase function. After incubation, the disks were removed and placed on a lawn of susceptible *E. coli* (ATCC 25922) to determine whether the test organisms degrade meropenem. Clearance zones were measured and interpreted according to the CLSI guidelines [17].

### Genotyping of β-Lactamases and Outer Membrane Proteins

The β-lactamase-encoding gene was selected through polymerase chain reaction (PCR). Genes encoding carbapenemases (IMP-1-type, VIM-2-type, NDM, KPC, KPC-2, GES, and OXA-48-like) and extended-spectrum β-lactamases (CTX-M-1-, CTX-M-9-, TEM-, and SHV-type), were assessed by PCR as previously described [19]. CPE isolates were examined for the presence of 16S ribosomal methyltransferases (armA, rmtA, rmtB, and rmtD) [20, 21], and quinolones (qepA, qnrA, qnrB, and qnrS), as previously described [22], and of genes encoding outer membrane proteins (ompK35 and ompK36) [23].

### MLST

PCR and sequencing of the amplified DNA fragments were performed for seven housekeeping genes: *adk*, *fumC*, *gyrB*, *icd*, *mdh*, *purA*, and *recA* of *E. coli* [24] and *gapA*, *infB*, *mdh*, *pgi*, *phoE*, *rpoB*, and *tonB* of *K. pneumoniae* [25]. Nucleotide sequences were compared to those available in the MLST database (http://mlst.warwick.ac.uk/mlst/dbs/Ecoli) to identify allelic numbers and STs.

### Bacterial Conjugation

Bacterial conjugation was conducted using the *E. coli* strain CPEc171209 and *Klebsiella pneumoniae* CPKp171210 strains as donors and sodium azide-resistant *E. coli* J53 strain as the recipient, following a standard agar mating method [26]. After overnight incubation at 37°C on brain-heart infusion agar (MB Cell, USA), transconjugants were selected on brain-heart infusion agar supplemented with 100 μg/ml sodium azide and 0.5 μg/ml imipenem.

### Whole-Genome Sequencing

Whole-genome sequencing of CPEc171209 and CPKp171210 was performed. Single-molecule real-time sequencing was performed using a PacBio RSII instrument (Pacific Biosciences, USA). Schematic diagrams of the multiple alignments of plasmids were generated manually by realigning the linear plasmid maps generated using the SnapGene Viewer software (http://www.snapgene.com/products/snapgeneviewer/). Prokka 1.11 (http://www.vicbioinformatics.com/software.prokka.shtml) was used for sequence annotation. ResFinder (https://cge.cbs.dtu.dk/services/ResFinder/), IS-Finder (https://isfinder.biotoul.fr/), Plasmid Finder (https://cge.cbs.dtu.dk/services/PlasmidFinder/), Virulence Factor Database (http://www.mgc.ac.cn/VFs/), Restriction-Modification Finder (https://cge.cbs.dtu.dk/services/Restriction-ModificationFinder/), Serotype Finder (https://cge.cbs.dtu.dk/services/SerotypeFinder/), Fim Typer (https://cge.cbs.dtu.dk/services/FimTyper/), CH Typer (https://cge.cbs.dtu.dk/services/CHTyper/), and TA Finder 1.0 (http://202.120.12.133/TAfinder/index.php) were used to identify resistance genes, insertion elements, replication origins, virulent elements, and toxin and antitoxin systems, respectively.

### Data Availability

GenBank accession numbers for the two sequenced genomes are WMHS01000001-WMHR01000006 (CPEc171209) and WMHR01000001-WMHR01000004 (CPKp171210).

## Results

### Antimicrobial Susceptibilities and Molecular Typing

The antimicrobial susceptibility profiles of the *E. coli* and *K. pneumoniae* strains are presented in Table 1. According to the antibiotic susceptibility profiles, both isolates were resistant to ampicillin, aztreonam, cefotaxime, ceftazidime, cefoxitin, ciprofloxacin, gentamicin, imipenem, meropenem, and ertapenem but susceptible to amikacin, tigecycline, and colistin. Carbapenemase susceptibility tests revealed that both isolates were resistant (Table 2). Furthermore, transconjugant strains displayed multidrug resistance (MDR) patterns during drug susceptibility assays, albeit with reduced cephalosporin and aminoglycoside resistance (Table 3). Differentiation tests for Phenotypic carbapenemase were positive for KPC production in both isolates. PCR of β-lactamase genes and sequence analysis of the resulting products supported the presence of *blaKPC-2* and *blaSHV-like* in both isolates. Also, *ompK36* was obliterated from CPKp171210.

### Sequencing and Annotation of CPEc171209

The *E. coli* strain CPEc171209 genome comprised 5,293,485-bp with a 4,787,633-bp chromosome and five plasmids. They harbored the virulence factors gad and lpfA as well as S83L and D87N substitutions in *gyrA*, an S80I substitution in *parC*, an S458A substitution in *parE*, and unknown mutations in *parC*, *pmrA*, *23S*, *16S_rsC*, *pmrB*, *16S_rsH*, and *16S_rsB*. The type II restriction enzyme *M.Eco*JA03PDcm, and *M.Eco*GVI genes were detected. CPEc171209 belonged to ST410, the serotype belonged to STO8, and H21 and its subtype belonged to fum C4 and fim H24, respectively. Furthermore, outer membrane proteins F and C were detected. Chromosomes included *mph (A)* for macrolide resistance. The 90,979-bp plasmid (pECBHS_9_3) and 85,870-bp plasmid (pECBHS_9_4) did not contain any acquired antimicrobial resistance factors.

**pECBHS_9_1 plasmid composition**. The pECBHS_9_1 plasmid consisted of a 188,153-bp circular DNA molecule with an average G+C content of 51.1% and 126 annotated open reading frames (ORFs).

**Fig. 1:**
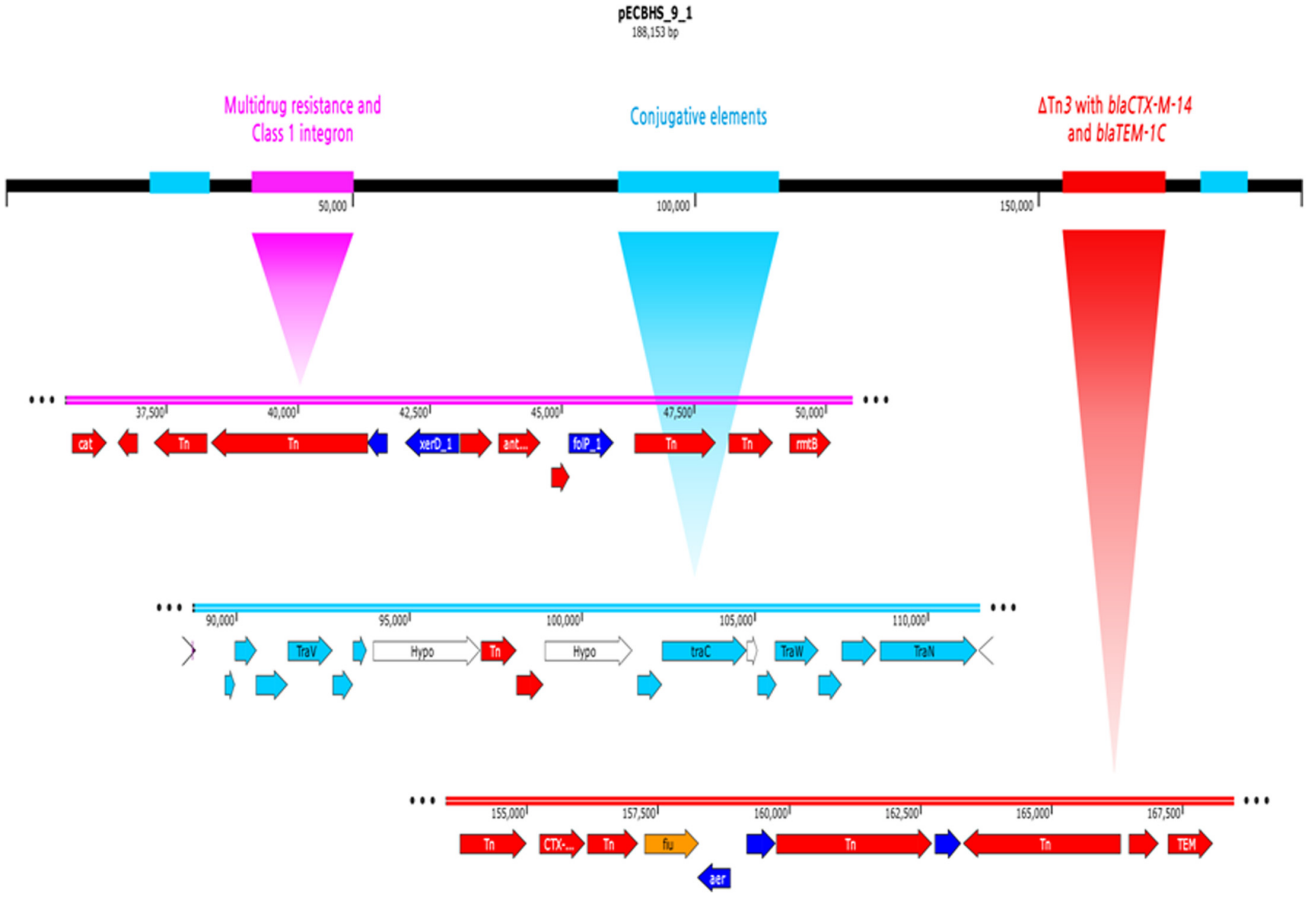
Linear map of pECBHS_9_1. The IncA/C2 plasmid (pECBHS_9_1) harbored *blaCTX-M-14* and *blaTEM-1C* for β-lactam resistance, *aadA5,* and *armA* for aminoglycoside resistance, *mph (A)* for macrolide resistance, *catA1* for phenicol resistance, *sul1,* and *sul2* for sulfonamide resistance, and *dfrA17* for trimethoprim resistance. The plasmid harbored Class 1 integrons containing a truncated Tn*3* transposon with *blaTEM-1C*. Key: Pink line, Multidrug resistance and Class 1 integron; red line, Tn*3* transposon; sky blue line, conjugative elements.

The IncA/C2 plasmid (pECBHS_9_1) carried *blaCTX-M-14* and *blaTEM-1C* for β-lactam resistance, *aadA5,* and *armA* for aminoglycoside resistance, *mph (A)* for macrolide resistance, *catA1* for phenicol resistance, *sul1* and *sul2* for sulfonamide resistance, and *dfrA17* for trimethoprim resistance. The plasmid harbored class 1 integrons with a truncated Tn3 transposon with *blaTEM-1C* (Fig. 1; Table 4).

**pECBHS_9_2 plasmid composition**. The incompatible pECBHS_9_2 plasmid contained three origins of replication for IncFIA, IncFIB, and IncFII groups and conjugal transfer (*tra* and *trb*). It also contained *aac (3)-IIa* for aminoglycoside resistance, *blaCTX-M-14* for β-lactam resistance, and *tet (a)* for tetracycline resistance. Along with drug resistance determinants, pECBHS_9_2 contained three toxin/antitoxin systems, including *iucA*, *iucB*, *iucC_1*, *iucC_2*, *iucD*, and *iutA*, associated with hydroxamate siderophore aerobactin synthesis.

**Fig. 2:**
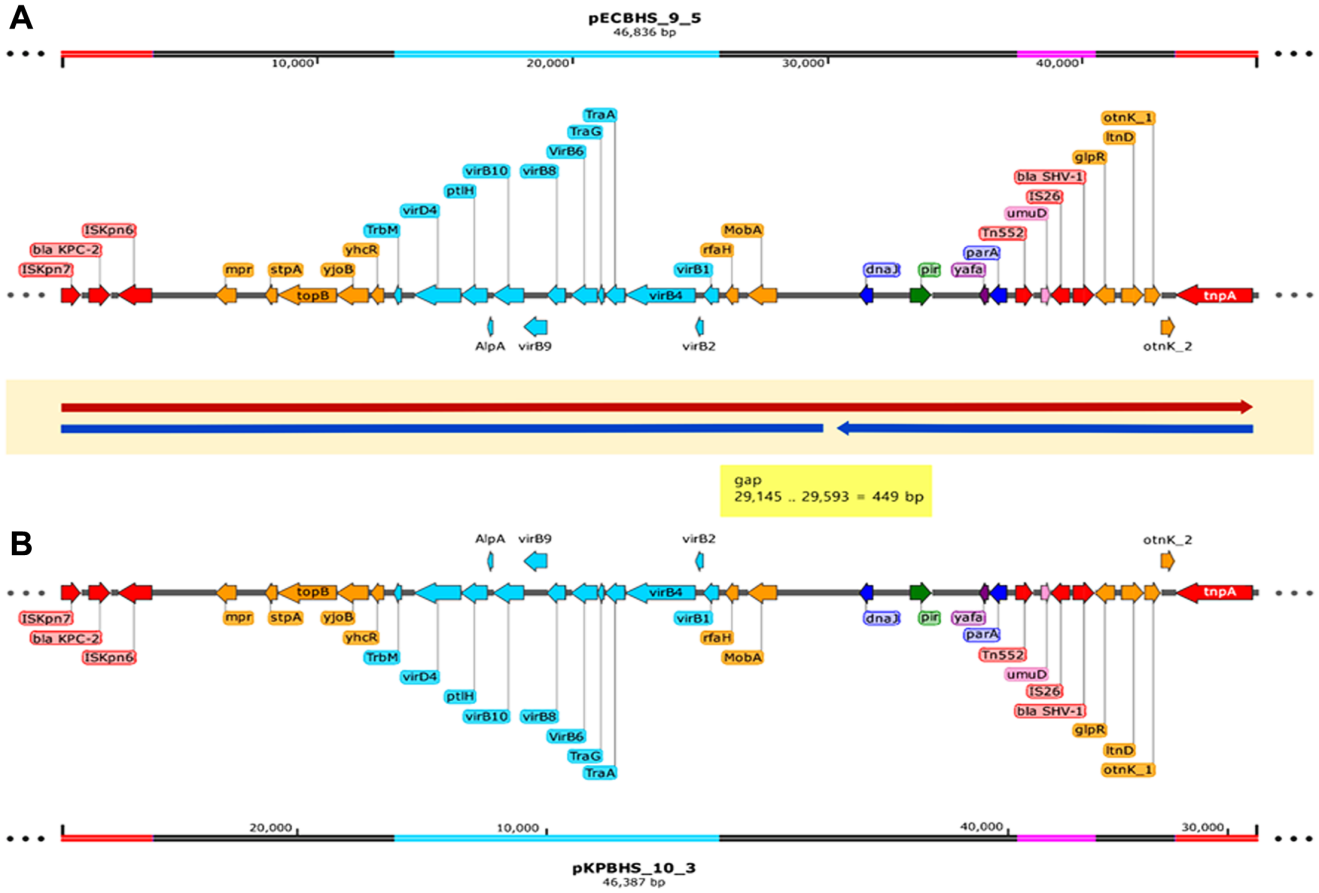
Comparison of the *blaKPC-2* regions of pECBHS_9_5 and pKPBHS_10_3. (**A**) Linear map of pECBHS_9_5 harboring ΔTn*4401a* with *blaKPC-2*, *blaSHV-182*, and conjugative elements. (**B**) Linear map of pKPBHS_10_3 harboring ΔTn*4401a* with *blaKPC-2*, *blaSHV-182*, and conjugative elements. The colored box indicates the gene. Most genes were well preserved but inverted. Genes are denoted by arrow colors based on the following gene function classification: green, plasmid replication; blue, plasmid stability; yellow, transcription and translation; sky blue, conjugative elements; pink, recombination and repair; red, antibiotic resistance; purple, other genes. Key: Pink line, *blaSHV-182*; red line, ΔTn*4401a* with *blaKPC-2*; sky blue line, conjugative elements.

**pECBHS_9_5 plasmid composition**. The 46,836-bp IncX3 plasmid (pECBHS_9_5) belonged to an incompatibility group and harbored *blaKPC-2* and *blaSHV-182* for β-lactam resistance. The circular DNA had a G+C content average of 48.0% and harbored 59 coding sequences and 25 annotated ORFs (Fig. 2). *blaKPC-2* was located on a Tn*4401* variant designated as “isoform a,” harboring a 99-bp deletion between *blaKPC* and *istB* [27]. DNA regions for plasmid replication (*repB*) and stability (*parAB* and *stpA*), origin of transcription and translation (*rfaH* and *yjoB*), and conjugal transfer (*tra* and *trb*) were observed, and the plasmid harbored *blaSHV-182* encoding a broad-spectrum β-lactamase (Fig. 2A).

### Sequencing and Annotation of CPKp171210

The *K. pneumoniae* strain CPKp171210 genome comprised 5,760,457-bp, with a 5,478,640-bp chromosome and three plasmids. The G+C content average was 57.3%. The 5,018 coding sequences comprised 86 tRNAs, 25 rRNAs, and 173 annotated ORFs. Unknown mutations in *ompK37*, *ompK36*, *acrR*, and *ramR* were observed. The chromosome encoded M.Kpn34618Dcm and type II restriction enzymes. Plasmid pKPBHS_10_1 M.EcoRII harbored a gene for Eco128I. The plasmid pKPBHS_10_2 contained restriction sites for type I restriction enzymes, S.Kpn1420II, and M.Kpn928I. CPKp171210 belonged to ST307, and the chromosome (CPKp171210) included *fosA* for fosfomycin resistance, *blaSHV-28* for β-lactam resistance, and *oqxA* and *B* for quinolone resistance.

**pKPBHS_10_1 plasmid composition**. The pKPBHS_10_1 plasmid comprised a 134,995-bp circular DNA with a G+C content of 52.0% on an average and 56 annotated ORFs. Plasmid pKPBHS_10_1 contained M.EcoRII and Eco128I type II restriction enzymes. pKPBHS_10_1 is an IncFIB(K) plasmid which harbored *blaOXA-1* for β-lactam resistance, *aac(6*′*)-Ib-cr* for aminoglycoside resistance, *qnrB1* for fluoroquinolone resistance, *catB3* for phenicol resistance, *tet(A)* for tetracycline resistance, and *dfrA14* for trimethoprim resistance. Plasmid pKPBHS_10_1 harbored CusCFBA genes for mediating resistance to copper and silver.

**pKPBHS_10_3 plasmid composition**. The 46,387-bp incompatible IncX3 plasmid (pKPBHS_10_3) harbored *blaKPC-2* and *blaSHV-182* for β-lactam resistance. This plasmid had a G+C content average of 48.0% and harbored 56 coding sequences and 38 annotated ORFs (Fig. 2B). *blaKPC-2* was located on a variant of Tn*4401*, called “isoform a,” that contained a 99-bp deletion between *blaKPC* and *istB*. DNA regions for plasmid replication (*repB*) and stability (*parAB* and *stpA*), origin of transcription and translation (*rfaH* and *yjoB*), and conjugal transfer (*tra* and *trb*) were detected, along with *blaSHV-182* encoding a broad-spectrum β-lactamase (Fig. 2B).

### Comparison of the Composition of pECBHS_9_5 and pKPBHS_10_3

**Fig. 3:**
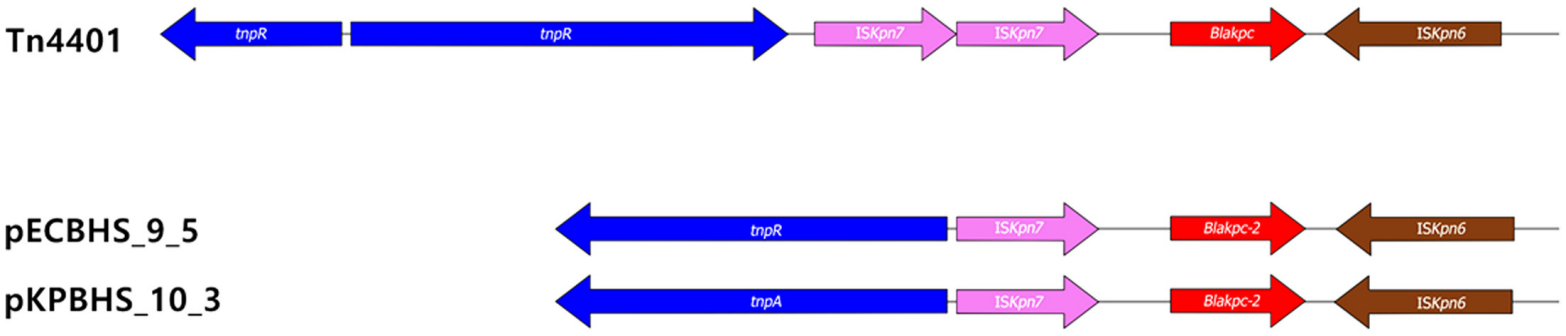
Comparison of wild type Tn4401 and truncated forms in the plasmids pECBHS_9_5 and pKPBHS_10_3. Transposon Tn4401 is presented with direct repeats in each plasmid. Arrows in pink box, IS*Kpn7*; arrows in brown box, IS*Kpn6*; blue arrows, transposase; red arrows, *blaKPC* gene.

Plasmid pECBHS_9_5 was 449 bp longer than plasmid pKPBHS_10_3. These two plasmids had 99% sequence similarity, except for the extra 449 bp in pECBHS_9_5. Alignment analyses between plasmid pKPBHS_10_3 and pECBHS_9_5 revealed 100% identity to the *blaKPC-2* allele (Fig. 2). Both plasmids were IncX3 incompatible plasmids harboring *blaKPC-2* and *blaSHV-182* β-lactam resistance genes. These plasmids had an average G+C content of 48.0% and could be transferred to the recipient *E. coli* J53 strain through surface conjugation (Table 3). *blaKPC-2* was located on the Tn*4401* “isoform a” variant (Fig. 3).

## Discussion

In this study, two clinical isolates, KPC-2 *E. coli* and KPC-2 *K. pneumoniae,* were obtained from rectal swabs. Recent studies on *E. coli* ST410 have suggested this stain as another successful pandemic extraintestinal pathogenic *E. coli* strain, with a lineage similar to that of ST131 [8]. Our results are largely concurrent with those of Roer et al. [10], with the *E. coli* (CPEc171209) ST410 strain belonging to the serotype O8:H21 containing antibacterial clade C4/H24 [10]. Roer *et al*. [10] reported that the *E. coli* ST410 lineage persists and causes recrudescent infections, such as hematological infections, in humans. ST410 has high worldwide prevalence and is widely distributed in the bloodstream, indicating that it can be transmitted between patients and cause hospital outbreaks [10]. *K. pneumoniae* (CPKp171210) ST307 harbors unknown mutations in *ompK36* and *ompK37*. In whole-genome sequencing analysis, these mutations were considered deletion mutations in DNA sequences. Isolates harboring *blaKPC* and expressing *ompK36* reportedly have lower carbapenem MICs. Furthermore, alterations in outer membrane proteins are reportedly associated with increased MICs of carbapenems [23]. Herein, both strains exhibited high or intermediate resistance to all tested antimicrobial agents, except for amikacin, tigecycline, and colistin. Resistance to a particular antibiotic can affect resistance to another antibiotic. For example, cross-resistance, such as those between cephalosporin and β-lactam, quinolone and methicillin- resistant *Staphylococcus aureus*, and macrolide and pneumococcal resistance, have been reported. MDR *E. coli* CPEc171209 and *K. pneumoniae* CPKp171210 also cause cross-resistance to various antibiotics (Table 1).

The genes responsible for conjugal transfer of the IncX3 plasmid include *tra/trb* clusters and *pil* genes coding the type IV pilus. The IncX3 type is a predominant plasmid associated with *blaNDM-1* [28] and occasionally harbors *blaKPC* [29], often accompanying a second plasmid including IncFIIκ and ColE types [30].

Plasmid pECBHS_9_5 of CPEc171209 harbored *blaKPC-2* bracketed by the Tn*3*-type transposon Tn*4401* to pKPBHS_10_3 of CPKp171210 (Figs. 2A and 2B). The *blaKPC-2* gene was located within a truncated Tn*4401* transposon; Tn*4401* harbors the *tnpA* gene encoding a transposase, the *tnpR* gene encoding a resolvase, and two insertion sequence elements, IS*Kpn7* and IS*Kpn6*, located at each end of the *blaKPC* gene [31]. Furthermore, both CPEc171209 and CPKp171210 harbored transconjugation factors and the Tn*3*-type transposon, potentially resulting in cross-infection and horizontal migration. Therefore, the risk of interspecies mobility and infection is high. Plasmid pECBHS_9_2 harbors genes encoding virulence factors and toxin/antitoxin systems, which results in the enhancement of bacterial fitness in human hosts and prolonged persistence [32]. KPC-2 *E.coli* and KPC-2 *K. pneumoniae* were continuously monitored by rectal swab detection over the course of 3 months until the patient died. Plasmids pKPBHS_10_1 and pKPBHS_10_2 harbor various genes related to the resistance against diverse kinds of antimicrobial agents and defend themselves through type I and II restriction enzymes. The pKPBHS_10_1 plasmid also harbors CusCFBA proteins mediating resistance to copper and silver through cation efflux. Gram-negative bacteria, including *E. coli* frequently use tripartite efflux complexes of the resistance- nodulation cell division superfamily transporters to pump out diverse toxic compounds [33, 34]. The efflux system CusCFBA is liable for removing biocidal Cu (I) and Ag (I) ions [35, 36], and the Cus determinant of gram- negative bacteria encodes CusCFBA proteins. CusA and CusB are essential for copper resistance, while CusC and CusF are required for overall resistance.

This study investigated a case of persistent infection in a patient because of interspecies migration of carbapenem-resistant *Enterobacteriaceae* caused by an *E. coli* ST410 strain. Molecular genetic evidence obtained herein reveals the nature of this occurrence and indicates the risk of ST410 *E. coli* infections. The emergence of KPC-producing *E. coli* is concerning because the blaKPC-2-bearing plasmid may result in interspecies propagation of resistance. *E. coli* ST410 represents a globally distributed lineage and is responsible for diverse antimicrobial resistance determinants, including extended-spectrum β-lactamases, pAmpCs, carbapenemases, and colistin resistance genes. Furthermore, the present results suggest that ST410 is a high-risk bacterium when it infects the host. Common sequences of virulence factors were observed in plasmids in ST410 CPEc171209 and in another high-risk group, ST307 CPKp1210, indicating the potential for cross-transmission, thus limiting treatment alternatives and maintaining long-term pathogenicity.

Carbapenem-resistant bacteria can undergo transgenic translocation and may spread rapidly via horizontal migration in patients. Together with its demonstrated high trainability among patients, *E. coli ST410* poses an elevated risk in clinical settings; therefore, it should be considered as a lineage with emerging “high-risk” clones and be closely monitored. Although the number of patients analyzed in this study was limited (1 patient), this study clearly elucidates the propagation pattern of KPC-producing *E. coli* (ST410) strains, which frequently occur in South Korea. Further investigation of the molecular genetics of KPC-producing *E. coli* (ST410) are needed.

## Figures and Tables

**Table 1:** Antimicrobial susceptibilities and epidemiological properties of KPC-producing *Enterobacteriaceae* isolates^a^

Isolate I.D	Specimen	Dates	MLST	*bla*KPC		Antibiotic susceptibility^b^						Carbapenemase differentiation test^c^		Porin Loss

			ST	Subtype	Bracketed by	MIC (mg/L)				Zone diameter (mm)		mCIM	eCIM	

						AK	GN	CST	TIG	AK	CST			
CPEc171209	Rectal	Dec 12 2017	410	*blaKPC-2*	*ΔTn4401a*	4	-	0.25	0.075	20	12	+	-	-
CPKp171210	Rectal	Dec 21 2017	307	*blaKPC-2*	*ΔTn4401a*	≤2	≤1	0.25	0.25	20	14	+	-	ompK36

a The breakpoints were applied according to the Clinical and Laboratory Standards Institute (CLSI) guidelines. Tigecycline susceptibility was confirmed by E-test (bioMérieux), and colistin susceptibility was confirmed by broth microdilution.

b Disk diffusion test results were interpreted according to CLSI guidelines. The results for colistin are not shown because of the lack of suggested breakpoints.

c The eCIM is only interpreted when the mCIM result is positive. In contrast to mCIM, when the eCIM result is interpreted, pinpoint colonies within the zone of growth inhibition around the meropenem disk incubated in the presence of EDTA should be ignored. An indeterminate mCIM result occurs when the zone size is 16–18mm, when the zone size is ≥19mm with pinpoint colonies in the zone of growth inhibition, or when carbapenemase production cannot be confirmed. An eCIM zone size of 16– 18mm with pinpoint colonies in the zone of growth inhibition is also considered a positive mCIM result.

Abbreviations: AK, amikacin; GN, gentamicin; CST, colistin; TIG, tigecycline; mCIM, modified carbapenem inactivation M, EDTA-modified carbapenem inactivation method.

**Table 2:** Carbepenem susceptibility profiles of KPC-producing *Enterobacteriaceae* isolates^a^.

Antibiotics	MIC (mg/L)				Zone diameter (mm)				Interpretation

	CPEc171209	CPKp171210	TCPEc171209	TCPKp171210	CPEc171209	CPKp171210	TCPEc171209	TCPKp171210	
IMP	≥16	≥16	≥16	≥16	0	0	0	0	R
MEM	-	-	-	-	0	0	0	0	R
EPM	≥8	≥8	≥8	4	0	0	0	0	R

a re-conjugation strains, and TCPEc171209 and TCPKp171210 are the conjugated strains. s: IMP, imipenem; MEM, meropenem; ETP, ertapenem.

**Table 3:** Antibiotic susceptibility profiles of KPC-producing *Enterobacteriaceae* isolates^a^.

Isolate I.D^b^	MIC (mg/L)																

	AMP	AMOX-CLA	TZP	CFZ	FOX	CTX	CAZ	FEP	ATM	ETP	IMP	AK	GN	CIP	SXT	TIG	CST
CPEc171209	≥32	≥32	≥128	≥64	≥64	≥64	≥64	≥64	≥64	≥8	≥16	4	≥16	≥4	≤0.5	0.075	0.25
CPKp171210	≥32	≥32	≥128	≥64	16	8	16	2	≥64	≥8	≥16	≤2	≤1	≥4	2	0.25	0.25
TCPEc171209	≥32	≥32	≥128	≥64	≥64	≥64	≥64	≥64	≥64	≥8	8	4	≥16	≥4	≤0.5	0.075	0.25
TCPKp171210	≥32	≥32	≥128	≥64	32	8	16	2	≥64	4	≥16	≤2	≤1	≥4	≤0.5	0.25	0.25

a The breakpoints were applied according to the Clinical and Laboratory Standards

b CPEc171209 and CPKp171210 are pre-conjug jugated strains.

Abbreviations: AMP, ampicillin; AMOX-CLA, amoxicillin/clavulanic acid; TZP, piperacillin/tazobactam; CFZ, cefazolin; FOX, cefoxitin; CTX, cefotaxime; CAZ, ceftazidime; FEP, cefepime; ATM, aztreonam; ETP, ertapenem; IPM, imipenem; AK, amikacin; GN, gentamicin; CIP, ciprofloxacin; ulfamethoxazole; TIG, tigecycline; CST, colistin. MIC; minimum inhibitory concentration.

**Table 4:** Antibiotic resistance genes and integrative conjugative elements in KPC-producing *Enterobacteriaceae* isolates.

Strain^a^	Plasmid		Antimicrobial resistance gene								Conjugants

	pKPC	Replicon	β-lactam	Aminoglycoside	Tetracycline	Macrolide	Phenicol	Trimethoprim	Sulphonamide	Quinolone	
CPEc171209	*blaKPC-2*		*blaTEM-1*, *blaCTX-M-1*, *blaCTX-M-9*, *blaSHV*	*armA*							+
TCPEc171209*	*blaKPC-2*		*blaTEM-1*, *blaCTX-M-1*, *blaCTX-M-9*, *blaSHV*	*armA*							
pECBHS_9_1		IncA/C2	*blaCTX-M-14*, *blaTEM-1C*	*aadA5*, *armA*		*mph(A)*	*catA1*	*dfrA17*	*sul1*, *sul2*		
pECBHS_9_2		IncFIA, IncFIB, IncFII	*blaCTX-M-14*	*aac(3)-IIa*	*tet(A)*						
pECBHS_9_5		IncX3	*blaKPC-2*, *blaSHV-182*								
CPKp171210	*blaKPC-2*		*blaTEM-1, blaCTX-M-1, blaSHV*	*rmtC*						*qnrB*	+
TCPKp171210*	*blaKPC-2*		*blaSHV*							*qnrB*	
pKPBHS_10_1		IncFIB(K)	*blaOXA-1*	*aac(6′)-Ib-cr*	*tet(A)*		*catB3*	*dfrA14*		*qnrB1*	
pKPBHS_10_3		IncX3	*blaKPC-2*, *blaSHV-182*								

a CPEc171209 and CPKp171210 are pre-conjugation strains, and TCPKp171210* are the conjugated strains. DNA sequencing was performed for strains that are underlined.

Whole-genome sequencing was performed for the indicated plasmids.
